# A Novel Isolator-Based System Promotes Viability of Human Embryos during Laboratory Processing

**DOI:** 10.1371/journal.pone.0031010

**Published:** 2012-02-29

**Authors:** Louise Hyslop, Nilendran Prathalingam, Lynne Nowak, Jeanette Fenwick, Steve Harbottle, Samantha Byerley, John Rhodes, Bruce Watson, Robin Henderson, Alison Murdoch, Mary Herbert

**Affiliations:** 1 Newcastle Fertility Centre, International Centre for Life, Newcastle upon Tyne, England, United Kingdom; 2 Institute for Ageing and Health, Newcastle University, Newcastle Fertility Centre, Centre for Life, Newcastle upon Tyne, England, United Kingdom; 3 Stockton Quality Control Laboratory, University Hospital of North Tees, Stockton-on-Tees, England, United Kingdom; 4 The Centre for Design Research, School of Design, Northumbria University, Newcastle upon Tyne, England, United Kingdom; 5 School of Mathematics and Statistics, Herschel Building, Newcastle University, Newcastle upon Tyne, England, United Kingdom; National Cancer Institute, United States of America

## Abstract

In vitro fertilisation (IVF) and related technologies are arguably the most challenging of all cell culture applications. The starting material is a single cell from which one aims to produce an embryo capable of establishing a pregnancy eventually leading to a live birth. Laboratory processing during IVF treatment requires open manipulations of gametes and embryos, which typically involves exposure to ambient conditions. To reduce the risk of cellular stress, we have developed a totally enclosed system of interlinked isolator-based workstations designed to maintain oocytes and embryos in a physiological environment throughout the IVF process. Comparison of clinical and laboratory data before and after the introduction of the new system revealed that significantly more embryos developed to the blastocyst stage in the enclosed isolator-based system compared with conventional open-fronted laminar flow hoods. Moreover, blastocysts produced in the isolator-based system contained significantly more cells and their development was accelerated. Consistent with this, the introduction of the enclosed system was accompanied by a significant increase in the clinical pregnancy rate and in the proportion of embryos implanting following transfer to the uterus. The data indicate that protection from ambient conditions promotes improved development of human embryos. Importantly, we found that it was entirely feasible to conduct all IVF-related procedures in the isolator-based workstations.

## Introduction

The establishment of a viable pregnancy capable of developing to term requires that a fertilized oocyte divides to form an embryo capable of giving rise to all cell types, including the extra-embryonic tissue (reviewed by [1]). In vitro fertilisation (IVF) typically involves fertilizing mature oocytes and culturing embryos in vitro for 2–6 days before selecting the best quality embryos for transfer to the uterus. Evidence from mouse and bovine studies indicates that in vitro cultured embryos show delayed development [2], [3] and reduced blastocyst quality [4] relative to their in vivo counterparts. The sparse information on the exact timing of implantation in humans [5] indicates the development of human embryos in vitro is also delayed. This may be linked to the findings that in vitro culture and manipulation is associated with stress-induced cellular responses [6], [7] epigenetic aberrations [8], [9], [10], and altered gene expression [11], [12], [13]. It is likely that exposure of oocytes and embryos to unphysiological conditions is an important contributory factor.

Laboratory processing during IVF typically involves the use of open-fronted microbiological (Class II) safety cabinets and stand-alone incubation chambers. Manipulations requiring removal of gametes and embryos from the incubator, therefore require exposure to ambient laboratory conditions which may result in deviations from physiological temperature and pH. This can alter cell physiology by disrupting fundamental cellular processes such as protein folding, enzyme activity, and assembly of the cytoskeleton [14]. For example, studies on human oocytes show that the meiotic spindle is sensitive to transient reductions in temperature [15], [16], [17] and that the prolonged exposure results in irreversible disruption of spindle organisation [15], [16]. This could contribute to the high levels of aneuploidy in in vitro cultured human embryos (reviewed by [18]).

The conventional practice is to culture human gametes and embryos in bicarbonate-buffered culture media under mineral oil in a CO_2_ enriched incubator with either ambient (21%) or reduced (5%) O_2_. Thus, exposure to ambient conditions can result in an upward drift in the pH of the culture medium. While it has been reported that alkaline conditions (pH 7.8–8.0) trigger a chloride-dependent response to maintain oocyte and embryo intracellular pH in the range of 7.0 to 7.4 [19], [20] it was found that the proportion of mouse and bovine embryos developing to the blastocyst declined as a function of increasing pH [21], [22], [23].

Volatile organic compounds (VOCs) [24] represent an additional potential hazard to gametes and embryos during laboratory processing. Reports from one group indicate that the level of VOCs in the IVF laboratory exceeded those in the outside air [25], [26]. Furthermore, VOC levels within incubators were elevated compared with those in the background laboratory [25], [26]. Amongst the hydrocarbon compounds detected were benzene, toluene, xylenes and styrene [25], which are known to be harmful to human health [27], [28]. Evidence from tissue culture cells indicates that m-xylene depletes endogenous thiols, which are scavengers of reactive oxygen species [29]. Moreover, exposure of mouse embryos to the VOCs acrolein and acetaldehyde, which are associated with building materials, was found to be detrimental to their development in vitro [26]. Together these findings indicate that embryos and gametes are potentially at risk from VOC-induced damage during laboratory processing.

To reduce the impact of environmental stresses due to physicochemical instability and exposure to chemical and microbial contaminants, we aimed to develop a means of protecting oocytes and embryos from exposure to ambient conditions throughout the IVF process. We designed an integrated chain of fully enclosed isolator-based [30], [31] workstations for manipulation of gametes and embryos. This approach enabled us to provide a controlled and monitored environment from the time oocytes are harvested from the ovary until embryos are transferred to the uterus. Analysis of clinical and laboratory data before and after the installation of the enclosed workstations indicates that protection from ambient conditions promotes increased cell proliferation and viability of human embryos.

## Methods

### Measurement of temperature and pH

Measurements of the pH and temperature of culture medium were obtained in 3 independent experiments using a calibrated pH meter (Jenway, UK) and thermocouple (Digitron, UK). Recordings were taken from tissue culture dishes placed on the microscope viewing area and containing 500 µl of pre-equilibrated bicarbonate buffered G1 (Vitrolife Ltd) culture medium overlaid with 700 µl of mineral oil. In the case of the open system, the dishes of media were pre-equilibrated in a BB6220 Heraeus CO_2_ incubator (Thermo Scientific). The enclosed system measurements were conducted following pre-equilibration in the custom built incubators (Vitrosafe Systems Ltd).

### Ovarian stimulation

Suppression of the hypothalamus/pituitary/ovarian feedback axis was achieved using GnRH analogue either as a nasal spray (800 µg daily; Nafarelin, Pfizer Ltd), or as sub-cutaneous injection (600 µg daily; Buserelin, Sanofi-Aventis Ltd). The superovulation regime consisted of daily subcutaneous injection of gonadotrophins (75–300 IU of the FSH and LH; Menopur, Ferring Pharmaceuticals) for 10–14 days followed by injection of hCG (5,000–10,000 IU; Pregnyl, Organon Laboratories Ltd) 38 hours before the scheduled time of oocyte harvest by transvaginal ultrasound guided aspiration. Progesterone pessaries (400 mg/day; Cyclogest, Actavis Ltd) were used for luteal support.

### In vitro fertilization and culture of human embryos

All culture media were purchased from Vitrolife (Gothenburg, Sweden). Oocyte retrieval was conducted using MOPS-buffered (G-MOPS) medium in the open-fronted system and bicarbonate-buffered (G-IVF) medium in the enclosed system. In both systems, oocytes for intra-cytoplasmic sperm injection (ICSI) were prepared by enzymatic removal of surrounding cumulus cells using hyaluronidase (Hyase, Vitrolife) in G-MOPS medium. Sperm injection was also preformed in G-MOPS medium. Injected oocytes were transferred to organ culture dishes containing bicarbonate-buffered G1 medium under mineral oil as described above. The bicarbonate-buffered medium G-Fert or G-IVF was used for fertilization by conventional IVF. Normally fertilized zygotes, which were identified by the presence of two pronuclei at 17–20 hours after addition of sperm, were transferred to bicarbonate-buffered G1 medium under mineral oil. The best quality embryos were selected for replacement based on morphological appearance and cell number on day 2 or day 3 after oocyte retrieval. Remaining embryos were cryopreserved, donated to research, or discarded. Embryos donated to research were cultured in G1/G2 media and monitored for development to the blastocyst stage at 5–7 days after oocyte retrieval. All procedures in the enclosed system were conducted in heated air, which except for cumulus cell removal and sperm injection, was enriched with 7% CO_2_.

### In vitro culture of mouse embryos

CD1 mice were kept on a 12 h light/dark cycle from 07:00 to 19:00 and naturally mated. Following detection of a copulatory plug, the mice were sacrificed and one oviduct was transferred to an open-fronted cabinet and the other oviduct to an enclosed isolator-based system. Pronuclear stage zygotes with surrounding cumulus cells were transferred to a hyaluronidase-containing medium (Hyase, Vitrolife) in either G-MOPS for samples processed in the open-fronted system, or in G1 embryo culture medium for samples in an enclosed research workstation. Following dissociation of the cumulus cells the zygotes were transferred to G1 under mineral oil for onward culture until the 8-cell stage when embryos were transferred to G2 medium.

### Blastocyst cell counts

Mouse (day 4.5–5.5 post coitum) and human (day 6–7 after oocyte retrieval) blastocysts were fixed using 4% PFA and were stained with the DNA dye Hoescht 33528 (Sigma, UK). Nuclei were visualized using an epi-fluorescence microscope (Nikon TE2000) fitted with an Apotome (Zeiss, Germany) or by confocal microscopy (Zeiss LSM 510 META). Nuclear counts were performed using Metamorph or Zeiss LSM 510 imaging software. We determined the accuracy of cell counting by different operators and imaging systems by performing a correlation to determine whether there were differences between counts (Fig. S1) and limits of agreement were deduced [32] by calculating 2 times the standard deviation (SD) of the differences between the 2 counts.

Research using human embryos was approved by the Newcastle and North Tyneside Research Ethics Committee and licensed by Human Fertilisation and Embryology Authority (HFEA). Written informed consent was obtained from all embryo donors.

### Comparison of clinical data

During the period when the new isolator equipment was being fitted and validated, our conventional equipment was transferred to temporary facilities so that the clinical service could continue. The effect of the enclosed system on treatment outcome was determined by comparing three cohorts of patients in 3 consecutive groups. Group 1 (n = 1213 cycles) was treated in our original laboratory using conventional open fronted cabinets; Group 2 (n = 619 cycles) was treated in a temporary laboratory using conventional equipment, and Group 3 (n = 798 cycles) was treated in the refurbished laboratory using the enclosed isolator-based system.

### Statistics

Logistic regression models were used to investigate factors associated with positive pregnancy outcomes, including group, age, and time trends. Chi-squared or 2 sample Student t-tests were used to test for significant differences. We applied moving average analysis of groups of 20 embryos to obtain trend information on blastocyst development. A correlation was carried out to determine whether there were differences between blastocyst nuclear counts performed by different operators.

## Results

### Design and implementation of a chain of enclosed workstations with incubators

**Figure 1:**
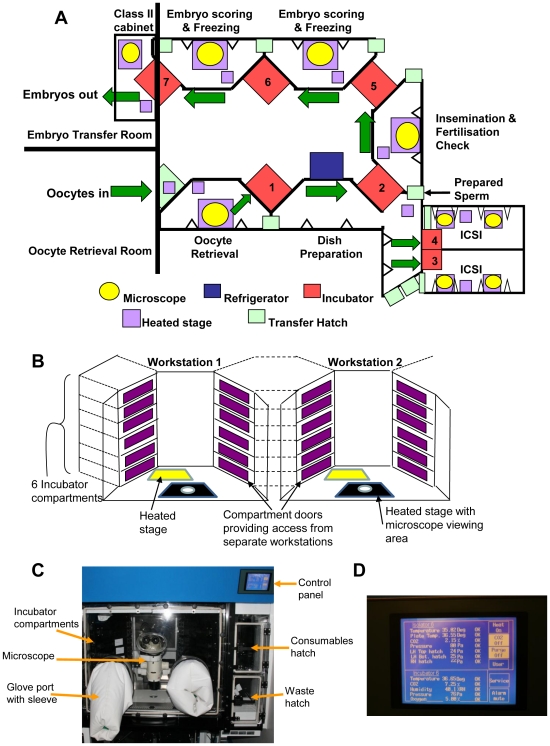
Layout of the enclosed workstations. (**A**) Schematic illustrating the layout of the enclosed workstations showing the workflow from the entry of oocytes into the system until the embryos are replaced in the uterus. The incubators act as pass-through hatches linking adjacent workstations. (**B**) Schematic diagram showing the layout of 2 enclosed workstations connected by the double-doored incubation chambers. (**C**) Image showing the main features of an enclosed workstation with a built-in stereomicroscope and incubators. There are separate hatches to allow introduction of consumables and removal of waste. (**D**) Image shows the control panel for setting the temperature of the incubator, workstation and hotplate and the %CO_2_ in the incubator and workstation. An alarm sounds when the environmental parameters deviate from the set-points.

We designed a chain of pressure-sealed enclosed workstations with integrated incubators and built-in microscopes for manipulation and culture of gametes and embryos (‘Laboratory Apparatus with Incubator’ patent application publication numbers: Europe EP1940548 A1, UK GB2433042, US US2010/0291664, Canada CA2628266; Fig. 1). The concept was based on isolator technology, which is conventionally used for the production of sterile pharmaceutical products [30], [31]. The chain of workstations was configured to link adjacent patient treatment rooms in which oocyte retrievals and embryo transfers are conducted (Fig. 1A). To avoid removing embryos from the enclosed system, we designed double-doored incubation chambers, which can be accessed from adjacent workstations (Fig. 1B). This enabled us to maintain oocytes and embryos within the enclosed environment from the time of oocyte harvest until transfer of embryos to the uterus. The incubators, which were designed with a dedicated air-supply, consist of 3–6 self-contained compartments to reduce the risk of cross-contamination between patient samples (Fig. 1B). Workstations were fitted with two interlocking ventilated hatches, to provide separate routes for transferring consumables into the cabinet and for removing waste materials (Fig. 1C). Each workstation was designed with a dedicated recirculating laminar air supply negatively pressurized with respect to the incubation chambers, and positively pressurized with respect to the transfer hatches. The air supply to the critical workarea, and to the incubators was passed and recirculated through HEPA filters to remove particulates, and through activated carbon filters to remove VOCs.

A major advantage of this system is that the supply air to work area and to the incubation chambers can be heated and enriched with CO_2_ providing environmental control during manipulation as well as during incubation (Fig. 1D). In addition to the standard temperature, humidity and CO_2_ controls, the incubators are fitted with O_2_ sensors to facilitate culture in reduced O_2_. All environmental sensors are linked to an alarm and recording system. Thus, each workstation was designed to provide a self-contained controlled physiological environment for manipulation and incubation of gametes and embryos.

Given the technical complexity of IVF and ICSI procedures, ergonomic considerations were paramount. As is the case in standard isolators [30], [31] operator access to the work area and incubators is via glove ports fitted with a double ring cuff system (Amercare Ltd, UK), which facilitates glove changing without breaking the pressure seal. Two prototype workstations were manufactured and tested extensively prior to manufacture of the complete interlinked system. Following initial trials the design was modified to improve operator access to the incubator compartments. After a period of approximately 2 weeks in which operators became accustomed to working in the enclosed environment, it was found to be entirely feasible to perform all procedures, including intracytoplasmic sperm injection (ICSI) and preimplantation genetic diagnosis (PGD), within the new system. The manufacture and installation of the system was controlled via an agreed validation master plan. Installation of the complete system was followed by a period of extensive testing for compliance with design specifications, and validation by an independent contractor.

### Stability of environmental conditions

**Figure 2:**
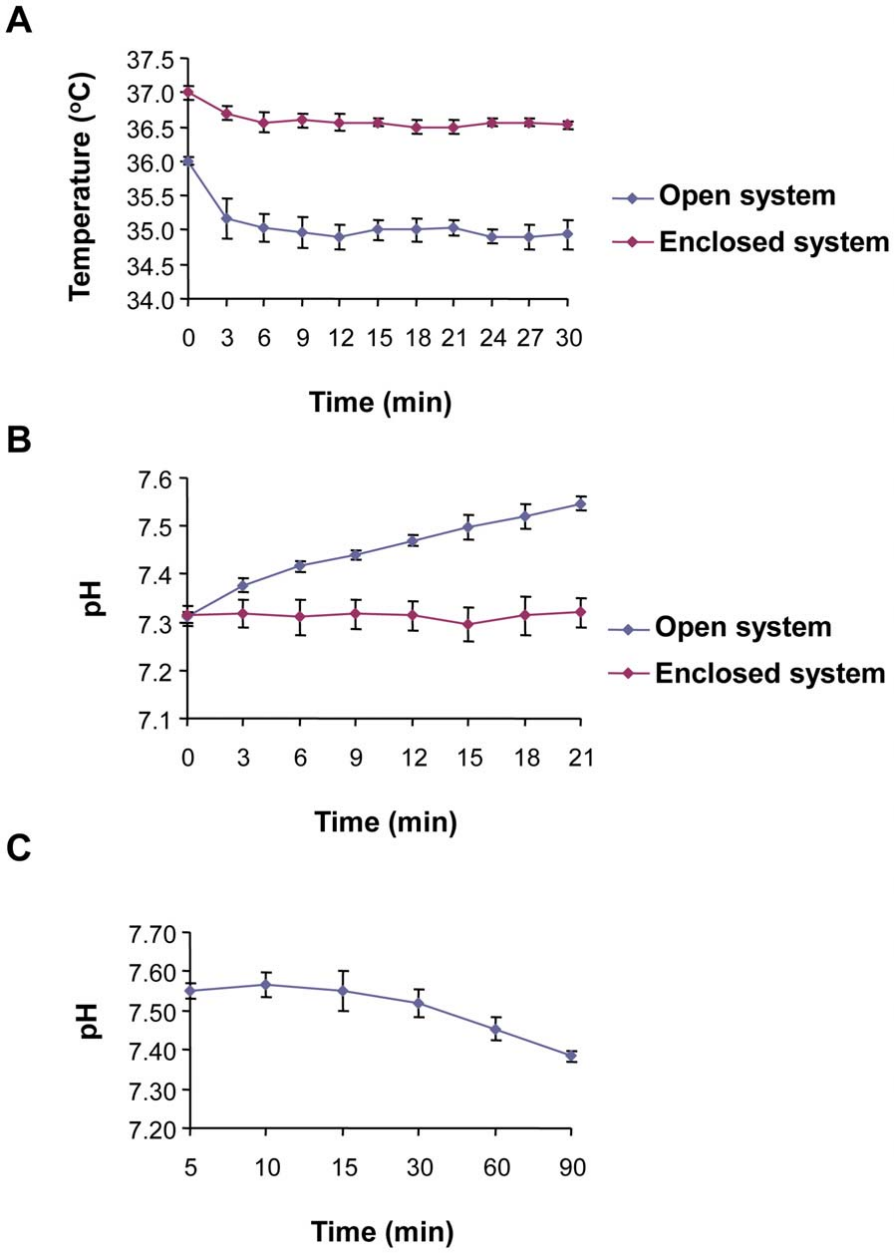
Comparison of temperature and pH stability between the enclosed workstations and conventional open-fronted cabinets. (**A**) A pre-equilibrated organ culture dish was removed from the incubator and placed on the viewing area of either the enclosed workstation (purple) or Class II hood (blue). Time point zero indicates the first measurement taken immediately after placing the dish on the viewing area. The temperature was then measured at 3-minute intervals. The temperature in the Class II cabinet dropped from 36°C±0.1 to 35.2°C±0.3 and then stabilised at 34.9–35.0°C±0.2. In the enclosed workstation, the starting temperature dropped from 37.0–36.7°C±0.1 and stabilised at 36.5–36.6°C±0.1. The lower starting temperature in the Class II cabinet is most likely due to heat loss during transfer from the incubator. In each case 3 independent experiments were conducted. (**B**) A pre-equilibrated organ culture dish was removed from the incubator and placed on the viewing area of either the enclosed workstation (purple) or Class II cabinet (blue). Time point zero indicates the first measurement taken immediately after placing the dish on the viewing area. There was a gradual increase in pH of the media in the Class II cabinet over the time course of the experiment. By contrast the pH of the media in the enclosed workstation remained in the range 7.30–7.32. In each case 3 independent experiments were conducted. (**C**) After completing the measurements for Figure 2B, the dish was returned to the incubator and pH measurements taken at 5 minute intervals for 90 minutes. The pH failed to return to 7.31 after 90 minutes in the incubator.

Our primary aim in developing the enclosed chain of workstations was to prevent fluctuations in temperature and pH during open manipulation of oocytes and embryos. We therefore compared stability of temperature and pH in the open and enclosed systems using conditions designed to simulate open manipulations of embryos and gametes in our laboratory. In three separate experiments we measured the temperature of 500 µl of pre-warmed culture medium with a 700 µl overlay of mineral oil in an organ culture dish, which was placed on the stereomicroscope viewing area of a conventional Class II cabinet fitted with a heated surface set at 37.4°C. We found that the temperature dropped from 36°C±0.1, to 35.2°C±0.3 within the first 3 minutes (Fig. 2A). By contrast, with an air-supply set-point of 35.0°C and a hotplate set-point of 37.2°C, the temperature of the culture medium in the isolator-based workstation was maintained at 36.5–36.6°C±0.2 for the duration of the experiment (Fig. 2A). The lower starting temperature of the culture medium in the Class II cabinet was most likely due to heat loss during transfer from the incubator.

The pH of pre-equilibrated culture medium increased from 7.31±0.01 to 7.38±0.02 in the first 3 minutes and increased further to 7.55±0.02 after 20 minutes in the Class II cabinet (Fig. 2B), whereas the pH within the isolator remained in the range of 7.30–7.33±0.04 for the duration of the experiment (Fig. 2B). The increase in pH observed in the Class II cabinet is modest compared with a previous report in which the pH increased from 7.05 to 8.0 within 5 minutes [33]. This is most likely due to the overlay of mineral oil causing a reduced rate of CO_2_ loss from the culture medium. However, following replacement in the incubator, the pH of the culture medium did not begin to drop for 15 minutes and failed to return to 7.31 during the 90 minute period of measurement (Fig. 2C). Thus, using a conventional system, the increase in pH although modest, remains elevated for a prolonged period following replacement in a CO_2_-controlled incubator.

Carbon filters were included in the design to protect against VOCs. Using a VOC meter with a detection limit of 0.1 ppm, we were unable to detect VOCs within the workstations or incubators. Background lab readings were also below the detection limit of the VOC meter. This indicates that under normal working conditions, VOCs are not a major concern in our laboratory environment. However, we believe that the carbon filters are important for protecting against the unforeseen events in which VOCs generated in the external environment might enter the laboratory air supply. Furthermore, carbon filtration of the re-circulated air in the enclosed system was deemed necessary to remove any internally generated VOCs.

### Development of embryos to the blastocyst stage in vitro

**Figure 3:**
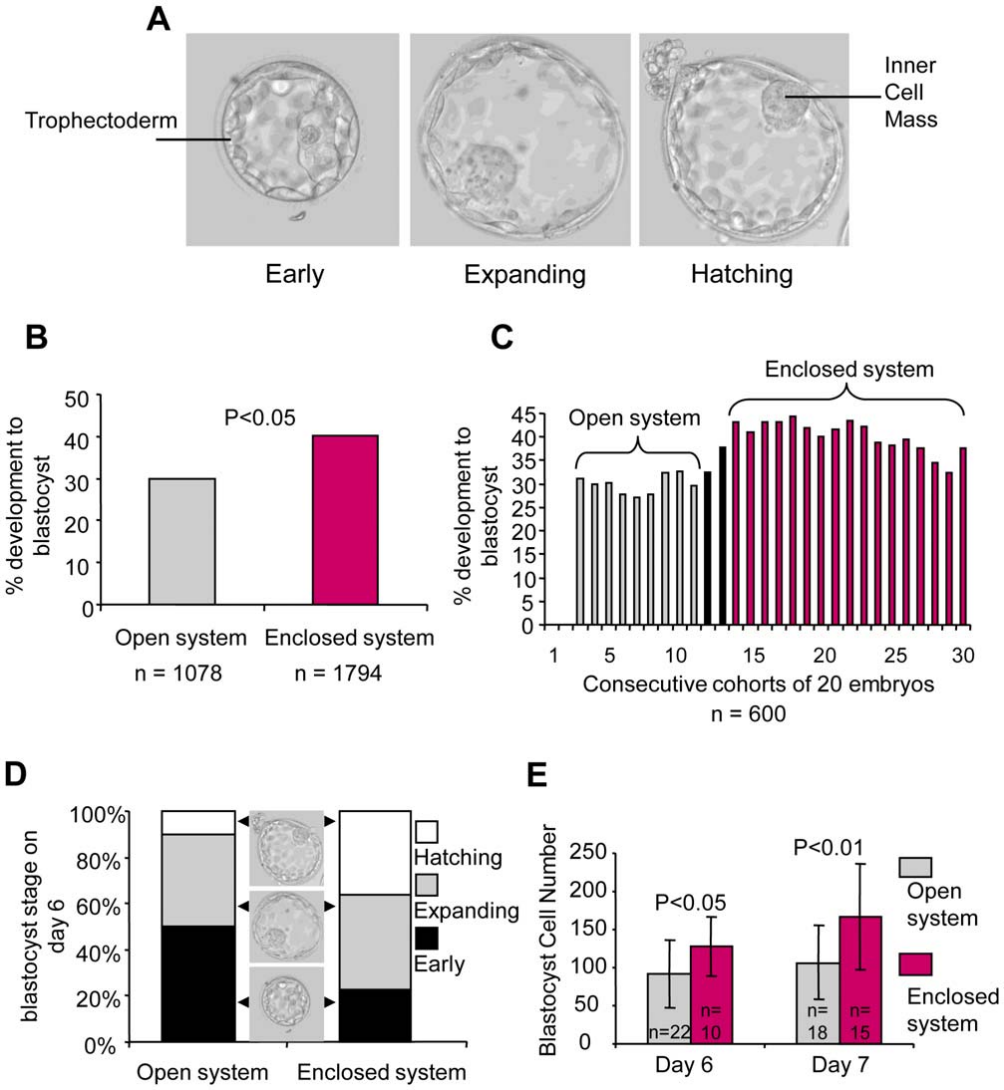
Comparison of development to the blastocyst stage between the enclosed workstations and conventional open-fronted cabinets. (**A**) Images show examples of early, expanded and hatched human blastocysts indicating the trophectoderm and the ICM. (**B**) Development of embryos to the blastocyst stage was compared between the open and enclosed systems. A significantly higher proportion of embryos developed to the blastocyst stage in the enclosed system (P<0.05). (**C**) Three point moving average of development to the blastocyst stage in consecutive cohorts of twenty embryos. The increase in the proportion of embryos developing to the blastocyst stage coincides with the switch from the open to the enclosed workstations. The two dark bars represent the intermediate period when the cohorts of 20 consisted of embryos cultured in the open and enclosed systems. (**D**) The proportions of blastocysts graded as early (black), expanded (grey) and hatched (white) on day 6. A significantly higher proportion of blastocysts had undergone hatching by day 6 after culture in the enclosed system compared to the open system. There was a corresponding reduction in the proportion of early blastocysts in the enclosed system (P<0.05). (**E**) Nuclear counts were performed to compare the total number of cells contained in blastocysts cultured to day 6 or 7 in the open system or enclosed system. The cell count of embryos cultured in the enclosed system was significantly higher on day 6 (128.3±38.6 *vs* 92.1±43.7; P<0.05) and day 7 (167.7±69.7 vs 106.7±48.8; P<0.01) compared with those cultured in the open system. See Figure S1A–C for an example of nuclear staining in a blastocyst and comparison of nuclear counts between different operators and imaging systems.

To determine whether the enclosed system promoted improved embryo development in vitro, we compared the proportion of embryos undergoing blastocyst formation before and after the installation of the enclosed system. Embryos used for this part of the study were allocated to research following completion of either IVF or ICSI treatment and were cultured from the time of fertilization until they reached the blastocyst stage either in the conventional system (n = 1078) or in the enclosed system (n = 1794). Blastocyst formation was determined by the appearance of a well defined trophectoderm layer surrounding a fluid filled blastocoel cavity and an inner cell mass (ICM) (Fig. 3A). We found that the overall proportion of embryos developing to the blastocyst stage by day 7 was 30% in the open system compared with 40.1% in the enclosed system (P<0.001; Fig. 3B). Analysis of consecutive cohorts of 20 embryos (n = 600) revealed that the timing of the increase in blastocyst formation coincided exactly with the change from the open to the enclosed culture system (Fig. 3C).

We also found that culture in the enclosed system was associated with accelerated progression of blastocyst development in vitro. Following the appearance of the blastocoel cavity, blastocysts undergo expansion before hatching from their zona pellucida (Fig. 3A). Comparison of the proportions of blastocysts that progressed to the expanded and hatched blastocyst stage by Day 6 showed a significantly higher proportion of hatched blastocysts (P<0.05) in the enclosed system (Fig. 3D). Moreover, comparison of blastocyst cell number based on nuclear counts of fixed blastocysts (Fig. S1A–C) revealed that embryos cultured to the blastocyst stage in the enclosed system contained significantly more cells on Day 6 and on Day 7 (P<0.05) compared with those cultured in the open system (Fig. 3E). It remains to be established whether the increased cell number in the enclosed system was a consequence of increased cell proliferation, reduced cell death, or a combination of the two.

**Figure 4:**
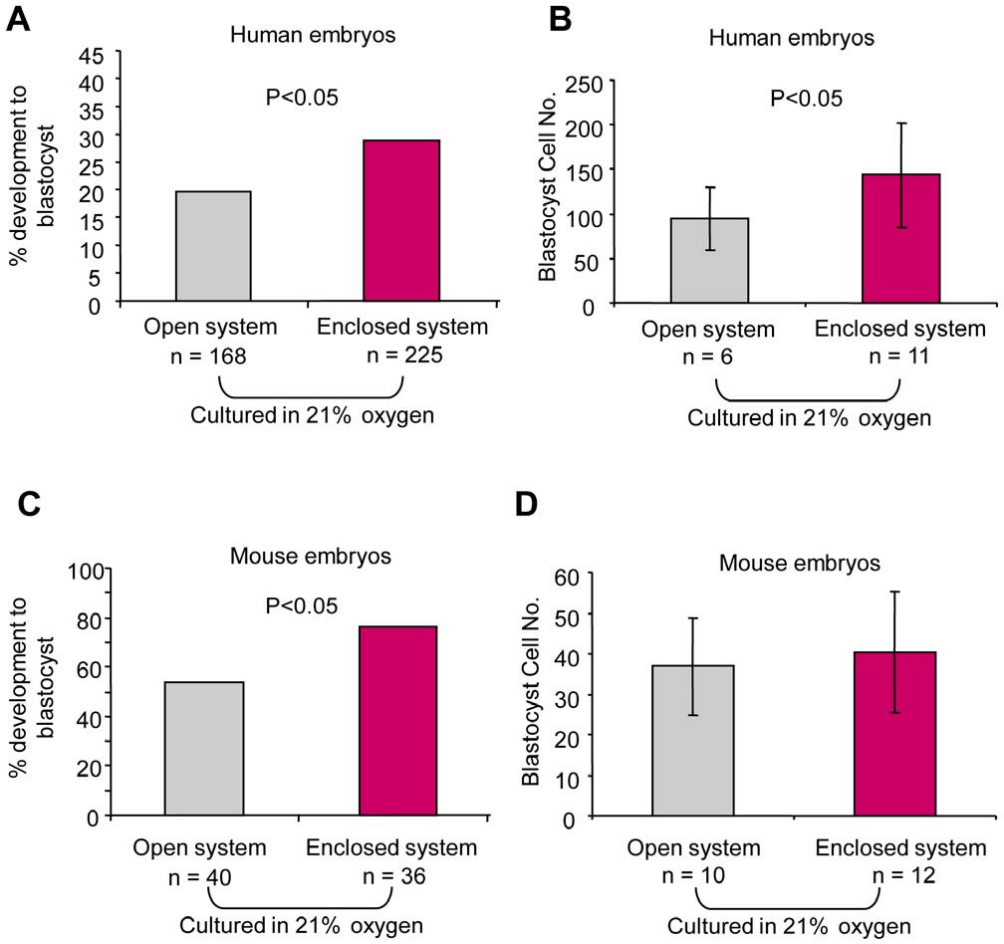
Effect of the enclosed system and 21% O_2_ tension on blastocyst development of embryos. (**A**) Development of human embryos to the blastocyst stage was significantly lower in the open system compared with the enclosed system when incubated in 21% O_2_; P<0.05. (**B**) Nuclear counts were performed to compare the total number of cells contained in human blastocysts cultured to day 6 in the open system or enclosed system in 21% O_2_. The cell count of human embryos cultured in the enclosed system in 21% O_2_ was significantly higher compared with those cultured in the open system (P<0.05). (**C**) A significantly higher proportion of mouse embryos developed from the 1 cell to the blastocyst stage in the enclosed system compared with the open system (P<0.05). Both sets of embryos were cultured in 21% O_2_. (**D**) Nuclear counts were performed to compare the total number of cells contained in mouse blastocysts cultured in the open system or enclosed system in 21% O_2_. The cell count of mouse embryos cultured in the enclosed system in 21% O_2_ was not significantly different compared with those cultured in the open system.

All embryos included in the above comparison were cultured in an atmosphere of 7% CO_2_, however, the O_2_ tension in the incubation chambers of the enclosed system was 5% compared with 21% in the open system. To gain insight into whether the improved blastocyst development was a consequence of reduced O_2_ tension during culture in the enclosed system, we analyzed blastocyst development of embryos (n = 225) cultured in the same gas phase (21% O_2_; 7% CO_2_) as the open system (n = 168). We found that the improved blastocyst formation was maintained under these conditions (P<0.05; Fig. 4A). The overall reduced blastocyst formation compared with the data presented in Figure 3B is likely due to the fact that this series consists entirely of ICSI embryos, which in our hands show reduced development to the blastocyst stage compared with IVF embryos [34]. Analysis of blastocysts in which nuclear staining was performed indicated that those cultured in the enclosed system contained significantly (P<0.05; Fig. 4B) more cells on Day 6 compared with those cultured in the open system. Taken together these findings indicate that the increased blastocyst formation and cell number of embryos cultured in the enclosed system was not solely a consequence of culturing embryos in reduced O_2_ tension.

In view of the constraints associated with working with human embryos, we performed a series of controlled experiments to test the effect of the enclosed system on the development of mouse embryos to the blastocyst stage. To control for variation between females we harvested pronucleate stage zygotes from the oviducts of individual females (n = 9) either in the enclosed system (n = 36 embryos) or in the open system (n = 40 embryos). Both sets of embryos were cultured using bicarbonate-buffered G1 medium in 21% O_2_, and were examined for equivalent periods (1–2 minutes) under the microscope at 22–24 hr intervals. Comparison of the proportions of embryos developing to the blastocyst stage showed significantly higher development in the enclosed system (P<0.05; Figure 4C), confirming our findings with human embryos. However, while the mouse blastocyst cell counts were slightly higher in the enclosed system (Fig. 4D), the difference was not significant. This may be due to the fact that the mouse embryos were cultured only from the 1-cell stage and therefore had reduced exposure to ambient conditions compared with human embryos.

### Clinical outcome data

**Figure 5:**
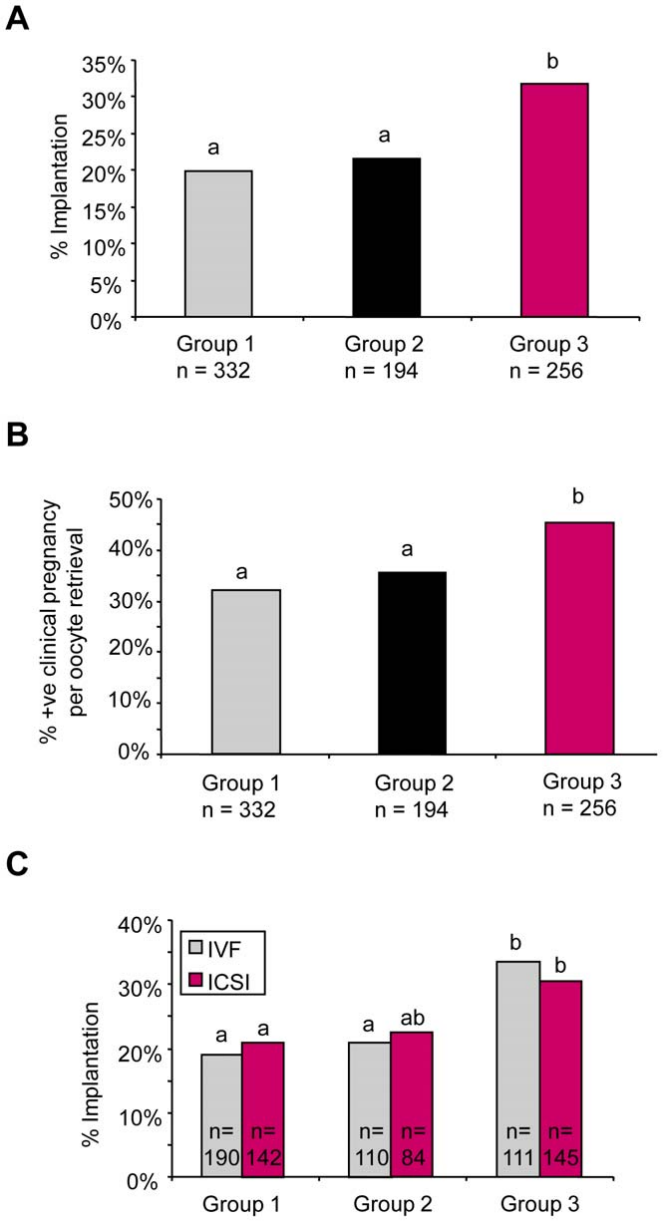
Effect of the enclosed system on IVF and ICSI clinical outcome measures. Comparison of the effect of the enclosed system on clinical outcome for consecutive groups of women aged ≤37 years with >10 ovarian follicles undergoing their first cycle of either IVF or ICSI treatment. Embryos from Groups 1 and 2 patients were cultured in the conventional open-fronted system but in different laboratories. Group 3 were conducted in the enclosed system. Letters indicate statistically significant difference. (**A**) The proportion of transferred embryos resulting in successful implantation, as determined by the presence of a fetal heartbeat at 7 weeks, was significantly higher in Group 3 (31.8%) compared with Group 1 (19.8%) or Group 2 (21.6%); P<0.05. (**B**) The clinical pregnancy rate per oocyte retrieval was 45.3% for Group 3 compared with 32.2% for Group 1 and 35.6 for Group 2; P<0.05. (**C**) Comparison of implantation rates in IVF and ICSI treatments in the open and enclosed system.

To determine whether improved development in vitro was matched by increased embryo viability in vivo, we compared clinical outcomes following transfer to the uterus of embryos cultured either in the open or enclosed systems. To control for variability between patient cohorts, we confined our analysis to couples undergoing their first treatment cycle in which the female partner was ≤37 years and had ≥10 follicles aspirated. Because treatments were conducted in a temporary laboratory during the 12 months before the introduction of the enclosed system, we sub-divided cycles processed in the open system into two groups: those processed in the original laboratory (Group 1; n = 332) and those processed in the temporary laboratory (Group 2; n = 194). Group 3 cycles (n = 256) were processed in the enclosed system. Comparison of the three groups revealed no difference in mean female age, mean number of oocytes harvested, mean number of fertilized zygotes, or mean number of embryos transferred (Table 1). However, the proportion of transferred embryos resulting in successful implantation, as determined by the presence of a fetal heartbeat at 7 weeks, was significantly higher in Group 3 compared with Group 1 (P<0.001) or Group 2 (P<0.001; Fig. 5A). Consistent with this, the clinical pregnancy rate per oocyte retrieval was 45.3% for Group 3 compared with 32.2% for Group 1 and 35.6 for Group 2 (P<0.05; Fig. 5B). Group 3 had a slightly higher proportion of ICSI cycles, however, this does not account for the improved outcome as the greatest improvement was observed in conventional IVF treatments (Fig. 5C).

**Table 1:** Comparison of patient profiles.

	Group 1 (n = 332)	Group 2 (n = 194)	Group 3 (n = 256)
IVF cycles (%)	57%	56.7%	43.4%
ICSI cycles (%)	43%	43.3%	56.6%
Mean female age (years ± SD)	31.6±3.9	31.4±4.0	31.0±3.8
Mean number eggs collected (±SD)	14.2±6.7	12.6±5.6	13.5±5.8
Mean number eggs normally fertilised (±SD)	8.0±4.8	8.0±4.3	7.7±4.2
Mean number embryos transferred (±SD)	1.91±0.36	1.96±0.22	1.90±0.36

Comparison of patient profiles between the open and enclosed systems for women ≤37 years with >10 follicles undergoing their first cycle of treatment. Groups 1 and 2 were processed in the open system but in different laboratories. Group 3 was processed in the enclosed system. There was no statistical difference between groups for any of the parameters measured.

**Figure 6:**
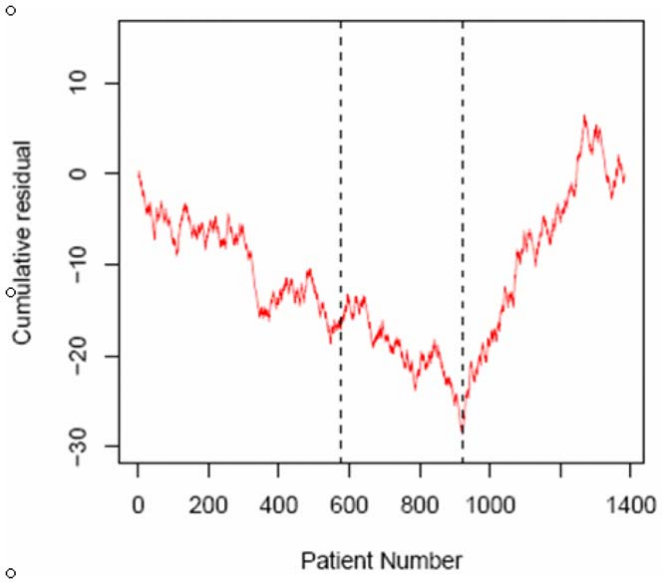
Logistic regression model comparing clinical pregnancy rates between the enclosed workstations and conventional open-fronted cabinets. Cumulative sum of residuals between observed clinical pregnancy rate and that fitted by an overall logistic regression model, plotted against time. During periods of stable performance the plot should have a constant slope: positive when performance is consistently better than average, negative for the opposite. The vertical lines mark the group boundaries. Regions with positive slopes indicate performance consistently better than average, regions with negative slopes indicate the opposite. The start of the third phase, group 3, coincides precisely with a change from a negative to positive slope and a statistically significant improvement in performance (p<0.001). There is no change in performance between the first and second phases or within any phase.

It could be argued that the improved outcomes in the enclosed system were attributable to a general improvement in assisted conception procedures during the period of this study. We tested this possibility by fitting a single logistic regression model to the data combined over all three groups, taking female age into account. We then tested for trends against time in the residuals between observed and model-fitted clinical pregnancy rate. If there are no time trends then a cumulative sum of residuals will have no systematic pattern. Periods of negative slope indicate periods of poor performance compared with the overall rate, and periods of positive slope indicate the opposite. The results showed consistently worse than overall average performance throughout the periods covered by Groups 1 and 2 and consistently better for Group 3 (Fig. 6). The start of Group 3 coincides precisely with a highly significant (p<0.001) change from a negative to positive slope. We therefore conclude that the improved treatment outcome was due to the beneficial effects of the enclosed system rather than to a general improvement in the outcome of assisted conception procedures.

## Discussion

Here we describe a novel enclosed isolator-based system designed to minimise the risk of environmental insult during laboratory processing for assisted conception treatment. We find that it is entirely feasible to conduct laboratory assisted conception procedures within this system. Measurements of the physicochemical parameters within the critical work area showed that the system is capable of maintaining stable temperature and pH. Analysis of the data before and after the introduction of the enclosed system indicates that it promotes increased development to the blastocyst in vitro. Moreover human embryos developing to the blastocyst stage showed accelerated development and contained significantly more cells than those cultured in the conventional system. Consistent with this we found that following transfer to the uterus, embryos created and cultured in the enclosed system resulted in a significantly higher implantation and clinical pregnancy rate compared with those cultured in the open system.

Our primary goal in developing the enclosed system was to minimize exposure of oocytes and embryos to pH and temperature fluctuations. In contrast to the enclosed system, pH measurements of bicarbonate-buffered culture medium with an oil overlay showed an upward drift to 7.55±0.02 in the open system followed by a prolonged (>90 min) recovery period upon return to the CO_2_-enriched incubator. However, the impact of this on embryo viability is unclear. Experiments using ion-sensitive fluorophores indicate that, in alkaline conditions, human embryos maintain intracellular pH in a narrow physiological range by means of a HCO_3_^−^/Cl^−^ exchange mechanism [19], [20]. The limited evidence on unfertilized human oocytes indicates that, in contrast to mouse and hamster oocytes [35], [36] they are equipped with a similar mechanism for regulation of intracellular pH [19], [20]. Nonetheless, it has been reported that development of mouse embryos to the blastocyst stage is negatively correlated with the pH of culture medium in the range we observed in the open system [21], [22].

Enrichment of the air supply to the workarea with CO_2_ also offers the possibility of dispensing with zwitterionic buffers such as HEPES and MOPS, which are commonly added to media used for procedures such as oocyte retrieval and ICSI, but may have cytotoxic effects [37]. Following the introduction of the enclosed system, we dispensed with the use of MOPS-buffered medium for oocyte retrieval. This may have contributed to the beneficial effects of the enclosed system. Conversely, our use of MOPS-buffered medium in ambient CO_2_ for sperm injection in the enclosed system, may have contributed to lower magnitude of the increase in implantation rates for ICSI compared with IVF treatment. Our ongoing work seeks to validate bicarbonate buffered-media for sperm injection and to determine whether the outcome of ICSI treatment can be further improved by eliminating MOPS-buffered medium from our ICSI procedures.

Evidence on the beneficial effects of culturing human embryos in reduced O_2_ is controversial. A recent meta-analysis indicates no benefit in terms of clinical outcome [38] and the evidence on human blastocyst formation is conflicting [39], [40], [41], [42]. Our main dataset on human blastocyst development (presented in Fig. 3) was obtained during a period when the enclosed system incubators were set to run at 5% O_2_. Subsequent data obtained from human embryos indicate that the improvement in blastocyst formation and cell number was maintained when embryos were cultured in a gas phase of 21% O_2_ in the enclosed system. Under these conditions we also observed increased mouse blastocyst formation. However, in contrast to human, the mouse blastocysts cell counts were equivalent between the two systems. This may be because the mouse embryos were fertilized in vivo and were therefore subjected to fewer manipulations. Thus, the detrimental effect of the open system was likely reduced compared with human embryos. Alternatively, the beneficial effects of the enclosed may have been countered by the sensitivity of mouse embryos to ambient O_2_ tension [43] used in this series of experiments.

It remains to be established whether the accelerated blastocyst development and increased cell number in the enclosed system is a consequence of a positive effect during early and/or late pre-implantation development. The increased incidence of implantation following transfer of embryos fertilized and cultured for 2–3 days in the enclosed system indicates that the benefits occur from the earliest stages of development. In support of this, it has been reported that delayed development of mouse embryos in vitro can be rescued by allowing embryos to undergo the first cell cycle in vivo [44]. This implies that events occurring during the first cell cycle are particularly susceptible to effects of in vitro manipulation, and that this has a negative impact on subsequent development. Thus, meaningful evaluation of enclosed culture systems should encompass the entire period of development from insemination onwards.

The physical constraint of performing technically demanding assisted conception procedures in an enclosed environment was a major concern during the design of this system. However, we found that following a two week period of becoming accustomed to working in the isolators, all procedures could be performed with ease. The ergonomic design of the system also took account of the need for all internal surfaces to be accessible for routine cleaning. The system can also be fumigated if required.

Among the additional advantages of the enclosed system, we find that the controlled environment is excellent for training inexperienced operators as it protects against potential negative effects of the longer manipulation times. In addition, the separate incubation compartments offer increased protection against cross-contamination between patients, which is especially important in cases of high-risk infections such as Hepatitis B and HIV. Furthermore, by providing a physical barrier between the operator and the critical workarea, the enclosed system offers increased operator protection during treatment of high-risk cases. It also offers the possibility of reduced risk of operator DNA contamination during embryo biopsy for pre-implantation diagnosis (PGD). This is important as PGD relies on detecting genetic defects by amplifying DNA from only one or two embryonic cells [45].

The use of carbon filters offers protection from external sources of VOCs, such as those generated during building renovations [26]. As the air is recirculated through carbon filters, VOCs generated within the work area and incubators [25], [26] can also be removed. We believe that this is crucial for preventing a build-up of VOCs within the enclosed work area.

In conclusion, we have shown that it is possible to perform laboratory processing for IVF and ICSI within an enclosed isolator-based environment and that this is associated with improved embryo quality in vitro and increased implantation following transfer to the uterus. By offering protection against external influences such as chemical pollutants, and protection from temperature and pH fluctuations, the system facilitates consistent and reproducible outcomes in assisted conception treatments.

## Supporting Information

Figure S1 **Analysis of the relationship of nuclear counts between operators and imaging systems.** Analysis of the relationship between nuclear counts performed by different operators and imaging systems was carried out to determine the accuracy of cell counting. A correlation was carried out to determine whether there were any differences between counts. As there was no difference, the limits of agreement were deduced (Bland and Altman 1999) by calculating 2 times the standard deviation (SD) of the differences between the 2 counts. Scatter diagrams of the difference between the counts (y-axis) and the average of 2 of the counts (x-axis) were plotted to visualize the data. (**A**) Brightfield image of a human blastocyst on left. Image on right shows z-projection of a nuclear stained human blastocyst. Nuclear staining was carried out using Hoescht 33528. The cell number was determined from single planes which have been used here to produce the z-projection. 50 µm scale bar indicated. (**B**) Analysis of the relationship of nuclear counts between operators. The difference between counts from two different researchers plotted against the average of the counts, the dotted lines represent 2 times the SD. (**C**) Analysis of the relationship of nuclear counts between imaging systems. The difference between counts using 2 different imaging systems plotted against the average of the counts, the dotted lines represent 2× the SD. **Ref:** Bland JM, Altman DG. (1999) Measuring agreement in method comparison studies. Statistical Methods in Medical Research 8, 135–160.
(TIF)

## References

[1] Cockburn K, Rossant J (2010). Making the blastocyst: lessons from the mouse.. J Clin Invest.

[2] Bowman P, McLaren A (1970). Cleavage rate of mouse embryos in vivo and in vitro.. J Embryol Exp Morphol.

[3] Plante L, King WA (1994). Light and electron microscopic analysis of bovine embryos derived by in vitro and in vivo fertilization.. J Assist Reprod Gen.

[4] Rizos D, Ward F, Duffy P, Boland MP, Lonergan P (2002). Consequences of bovine oocyte maturation, fertilization or early embryo development in vitro versus in vivo: implications for blastocyst yield and blastocyst quality.. Mol Reprod Dev.

[5] Buster JE, Bustillo M, Rodi IA, Cohen SW, Hamilton M (1985). Biologic and morphologic development of donated human ova recovered by nonsurgical uterine lavage.. Am J Obstet Gynecol.

[6] Leese HJ, Donnay I, Thompson JG (1998). Human assisted conception: a cautionary tale. Lessons from domestic animals.. Hum Reprod.

[7] Xie Y, Liu J, Proteasa S, Proteasa G, Zhong W (2008). Transient stress and stress enzyme responses have practical impacts on parameters of embryo development, from IVF to directed differentiation of stem cells.. Mol Reprod Dev.

[8] Young LE, Fernandes K, McEvoy TG, Butterwith SC, Gutierrez CG (2001). Epigenetic change in IGF2R is associated with fetal overgrowth after sheep embryo culture.. Nat Genet.

[9] Maher ER, Afnan M, Barratt CL (2003). Epigenetic risks related to assisted reproductive technologies: epigenetics, imprinting, ART and icebergs?. Hum Reprod.

[10] Rivera RM, Stein P, Weaver JR, Mager J, Schultz RM (2008). Manipulations of mouse embryos prior to implantation result in aberrant expression of imprinted genes on day 9.5 of development.. Hum Mol Genet.

[11] Niemann H, Wrenzycki C (2000). Alterations of expression of developmentally important genes in preimplantation bovine embryos by in vitro culture conditions: implications for subsequent development.. Theriogenology.

[12] Morgan HD, Jin XL, Li A, Whitelaw E, O'Neill C (2008). The culture of zygotes to the blastocyst stage changes the postnatal expression of an epigentically labile allele, agouti viable yellow, in mice.. Biol Reprod.

[13] Kues WA, Sudheer S, Herrmann D, Carnwath JW, Havlicek V (2008). Genome-wide expression profiling reveals distinct clusters of transcriptional regulation during bovine preimplantation development in vivo.. Proc Natl Acad Sci U S A.

[14] Swain JE (2010). Optimizing the culture environment in the IVF laboratory: impact of pH and buffer capacity on gamete and embryo quality.. Reprod Biomed Online.

[15] Pickering SJ, Braude PR, Johnson MH, Cant A, Currie J (1990). Transient cooling to room temperature can cause irreversible disruption of the meiotic spindle in the human oocyte.. Fertil Steril.

[16] Almeida PA, Bolton VN (1995). The effect of temperature fluctuations on the cytoskeletal organisation and chromosomal constitution of the human oocyte.. Zygote.

[17] Wang WH, Meng L, Hackett RJ, Odenbourg R, Keefe DL (2001). Limited recovery of meiotic spindles in living human oocytes after cooling-rewarming observed using polarized light microscopy.. Hum Reprod.

[18] Mantzouratou A, Delhanty JD (2011). Aneuploidy in the human cleavage stage embryo.. Cytogenet Genome Res.

[19] Phillips KP, Léveillé MC, Claman P, Baltz JM (2000). Intracellular pH regulation in human preimplantation embryos.. Human Reprod.

[20] Dale B, Menezo Y, Cohen J, DiMatteo L, Wilding M (1998). Intracellular pH regulation in the human oocyte.. Hum Reprod.

[21] Brinster RL (1965). Studies on the development of mouse embryos in vitro. I. The effect of osmolarity and hydrogen ion concentration.. J Exp Zool.

[22] John DP, Kiessling AA (1988). Improved pronuclear mouse embryo development over an extended pH range in Ham's F-10 medium without protein.. Fertil Steril.

[23] Lane M, Bavister BD (1999). Regulation of intracellular pH in bovine oocytes and cleavage stage embryos.. Mol Reprod Dev.

[24] Goldstein AH, Galbally IE (2007). Known and unexplored organic constituents in the Earth's atmosphere.. Environmental Science and Technology.

[25] Cohen J, Gilligan A, Esposito W, Schimmel T, Dale B (1997). Ambient air and its potential effects on conception in vitro.. Hum Reprod.

[26] Hall J, Gilligan A, Schimmel T, Cecchi M, Cohen J (1998). The origin, effects and control of air pollution in laboratories used for human embryo culture.. Hum Reprod.

[27] Caprino L, Tonga G (1998). Potential health effects of gasoline and its constituents.. Environ Health Perspect.

[28] Molhave L (1991). Volatile organic compounds, indoor air quality and health.. Indoor Air.

[29] Coleman CA, Hull BE, McDougal JN, Rogers JV (2003). The effect of m-xylene on cytotoxicity and cellular antioxidant status in rat dermal equivalents.. Toxicology Letters.

[30] Tillett L (1999). Barrier isolators as an alternative to a cleanroom.. American Journal of Health-System Pharmacy.

[31] Midcalf B, Phillips WM, Neiger JS, Coles TJ (2004). Pharmaceutical Isolators..

[32] Bland JM, Altman DG (1999). Measuring agreement in method comparison studies.. Statistical Methods in Medical Research.

[33] Veraitch FS, Scott R, Wong JW, Lye GJ, Mason C (2008). The impact of manual processing on the expansion and directed differentiation of embryonic stem cells.. Biotechnol Bioeng.

[34] Griffiths TA, Murdoch AP, Herbert M (2000). Embryonic development in vitro is compromised by the ICSI procedure.. Hum Reprod.

[35] Lane M, Baltz JM, Bavister BD (1999). Bicarbonate/chloride exchange regulates intracellular pH of embryos not oocytes of hamster.. Biol Reprod.

[36] Phillips KP, Baltz JM (1999). Intracellular pH regulation by HCO_3_
^−^/Cl^−^ exchange is activated during early mouse zygote development.. Dev Biol.

[37] Swain JE (2010). Optimizing the culture environment in the IVF laboratory: impact of pH and buffer capacity on gamete and embryo quality.. Reprod Biomed Online.

[38] Sobrinho DBG, Oliveira JB, Petersen CG, Mauri AL, Silva LF (2011). IVF/ICSI outcomes after culture of human embryos at low oxygen tension: a meta-analysis.. Reprod Biol Endocrinol.

[39] Kea B, Gebhardt J, Watt J, Westphal LM, Lathi RB (2007). Effect of reduced oxygen concentrations on the outcome of in vitro fertilization.. Fertil Steril.

[40] Dumoulin JC, Meijers CJ, Bras M, Coonen E, Geraedts JP (1999). Effect of oxygen concentration on human in-vitro fertilization and embryo culture.. Hum Reprod.

[41] Kovacic B, Vlaisavljević V (2008). Influence of atmospheric versus reduced oxygen concentration on development of human blastocysts in vitro: a prospective study on sibling oocytes.. Reprod Biomed Online.

[42] Ciray HN, Aksoy T, Yaramanci K, Karayaka I, Bahceci M (2009). In vitro culture under physiologic oxygen concentration improves blastocyst yield and quality: a prospective randomized survey on sibling oocytes.. Fertil Steril.

[43] Davidson A, Vermesh M, Lobo RA, Paulson RJ (1988). Mouse embryo culture as quality control for human in vitro fertilization: the one-cell versus the two-cell model.. Fertil Steril.

[44] Karagenc L, Sertkaya Z, Ciray N, Ulug U, Bahçeci M (2004). Impact of oxygen concentration on embryonic development of mouse zygotes.. Reprod BioMed Online.

[45] Harton GL, Magli MC, Lundin K, Montag M, Lemmen J (2011). ESHRE PGD Consortium/Embryology Special Interest Group–best practice guidelines for polar body and embryo biopsy for preimplantation genetic diagnosis/screening (PGD/PGS).. Hum Reprod.

