# Supplementary figures and images for: A Novel Isolator-Based System Promotes Viability of Human Embryos during Laboratory Processing
Source: PLoS One. 2012 Feb 29;7(2):e31010. doi: 10.1371/journal.pone.0031010 (PMC3290619; doi:10.1371/journal.pone.0031010)

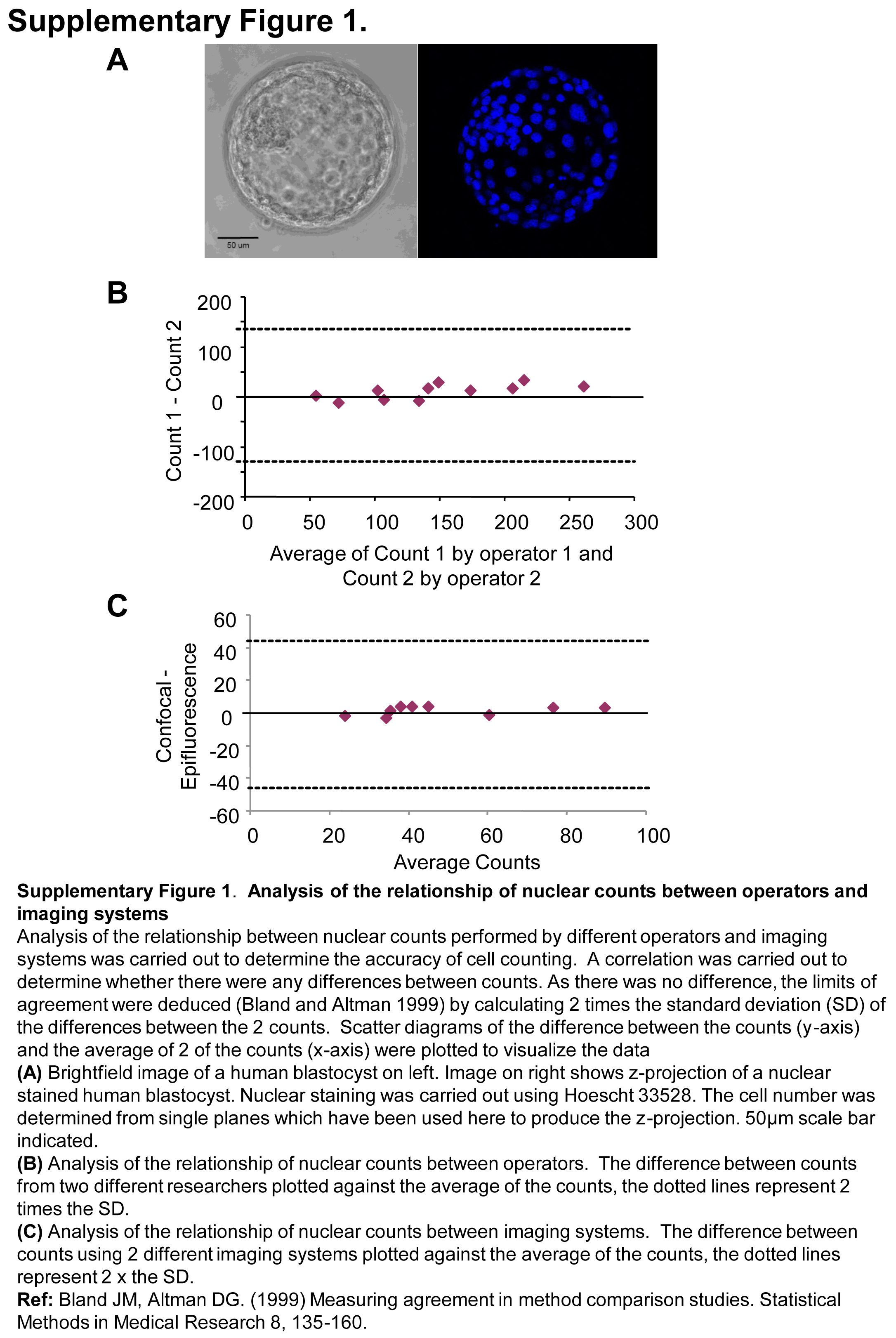

Supplement: Figure S1 — Analysis of the relationship of nuclear counts between operators and imaging systems. Analysis of the relationship between nuclear counts performed by different operators and imaging systems was carried out to determine the accuracy of cell counting. A correlation was carried out to determine whether there were any differences between counts. As there was no difference, the limits of agreement were deduced (Bland and Altman 1999) by calculating 2 times the standard deviation (SD) of the differences between the 2 counts. Scatter diagrams of the difference between the counts (y-axis) and the average of 2 of the counts (x-axis) were plotted to visualize the data. (A) Brightfield image of a human blastocyst on left. Image on right shows z-projection of a nuclear stained human blastocyst. Nuclear staining was carried out using Hoescht 33528. The cell number was determined from single planes which have been used here to produce the z-projection. 50 µm scale bar indicated. (B) Analysis of the relationship of nuclear counts between operators. The difference between counts from two different researchers plotted against the average of the counts, the dotted lines represent 2 times the SD. (C) Analysis of the relationship of nuclear counts between imaging systems. The difference between counts using 2 different imaging systems plotted against the average of the counts, the dotted lines represent 2× the SD. Ref: Bland JM, Altman DG. (1999) Measuring agreement in method comparison studies. Statistical Methods in Medical Research 8, 135–160. (TIF) [file pone.0031010.s001.tif]
